# A dual-matrix technique for direct composite veneers using liquid dam and pull-through contouring: a case report

**DOI:** 10.3389/fdmed.2026.1915125

**Published:** 2026-07-30

**Authors:** Mohammed Haji, Rand Saman Jadid, Daban Nariman, Louis Hardan, Rim Bourgi

**Affiliations:** 1 Department of Conservative Dentistry, College of Dentistry, University of Sulaimani, Sulaymaniyah, Kurdistan Region, Iraq; 2 Department of Dentistry, Komar University of Science and Technology, Sulaymaniyah, Kurdistan Region, Iraq; 3 Private Practitioner Dental Practice, Bakhtiari, Sulaymaniyah, Kurdistan Region, Iraq; 4 Department of Restorative and Esthetic Dentistry, Faculty of Dental Medicine, Saint-Joseph University of Beirut, Beirut, Lebanon

**Keywords:** case report, diastema closure, direct composite veneers, dual-matrix technique, liquid dam, minimally invasive dentistry, palatal shell, pull-through technique

## Abstract

**Background:**

Direct composite veneers for multiple anterior diastemas still present a challenge for a predictable palatal contour and controlled interproximal emergence profiles for interproximal emergence. Conventional matrices are not positionally stable and do not allow for morphological control, thereby leading to a technique-dependent procedure with the risk of inter-tooth asymmetry.

**Purpose:**

To present a dual matrix technique using liquid dam stabilization and a pull-through interproximal contouring while placing direct composite veneers in multiple anterior diastemas.

**Technique and case description:**

A 22-year-old female patient presented with bilateral peri-central diastemas (between teeth 12–11 and 21–22) and required a full aesthetic rehabilitation. The 2D-Digital Smile Design template was helpful for treatment planning. Composite veneers were placed on teeth 15 to 25. The isolation was performed using a rubber dam, and a palatal shell was fabricated using a transparent matrix modified extraorally and stabilized using liquid dam material. A pre-curved celluloid matrix was pulled from the facial to the palatal surface to form the interproximal forms and contact points. Composite resin was placed incrementally in dentin and enamel layers. The 10 restorations were evaluated at baseline (one week after the procedure) and at 12 months by both the modified United States Public Health Service (USPHS) and Fédération Dentaire Internationale (FDI) clinical performance grading criteria.

**Results:**

All restorations showed Alpha (USPHS)/FDI Score 1 criteria for all parameters examined at baseline. Marginal integrity, marginal adaptation, surface texture, color match, postoperative sensitivity, secondary caries, and tooth integrity were all Alpha/FDI Score 1 at the 12-month follow-up. Mild staining was found at three interproximal sites (15%) and marginal discoloration at one restoration (10%) (Bravo/FDI Score 2–3). Two restorations (20%) had a modest loss in surface luster, and one restoration (10%) had mild gingival marginal redness (both Bravo/FDI Score 2). One restoration had a localized incisal chip graded Bravo (USPHS)/FDI Score 3, which was successfully corrected chairside. No secondary caries or other biological problems were noted.

**Conclusions:**

The dual-matrix technique is a conservative single-visit procedure for the closure of anterior diastemas that can improve morphological control and reduce technique sensitivity. Concurrent use of USPHS and FDI criteria enabled comprehensive exploration and direct comparison with current studies.

## 1 Introduction

One of the major challenges in restorative dentistry is providing the least invasive and most esthetically pleasing anterior restorations. Although ceramic veneers have a 10-year failure rate of 2.8%, direct composite veneers are still popular because they are less invasive, reversible, preserve tooth structure, and require little or no preparation (1–3).

A systematic review found a pooled mid-term survival of 88% for direct composite veneers after a mean follow-up of 24 to 97 months (4). Longitudinal studies give a somewhat lower survival rate of 80.1% over 3.5 years with an annual failure rate of 4.9% on vital teeth (3). Randomized controlled trials show no significant short-term survival difference (2–3 years) when tested composites veneers in comparison to ceramics (5, 6), while composites display more surface change and marginal stain over time (7). The ceramic has a better survival rate on an enamel substrate, which can also be true for composites (8). In addition, anterior composites had better survival rates than posteriors in full-mouth rehabilitation, according to a previous study (9). Biocompatibility evaluations show that modern composite materials are very biocompatible and have very low cytotoxicity (10).

Differences in the fabrication technique also affect the esthetic effect. Spectrophotometric investigation revealed a clinically significant color difference between direct-indirect composite veneers and direct composite veneers, while visual clinical examination could not be detected, highlighting the fact that instrumental differences may not always be clinically evident (11).

Attention has shifted from material performance to operative technique, where computerized workflows, virtual treatment planning, and three dimensional (3D)-printed models improve reproducibility, and custom putty matrices or clear silicone stents permit precise replication (12, 13). Many techniques have been advocated. Direct-indirect veneers offer enhanced physical properties over freehand techniques, while prefabricated systems reduce treatment time for single-visit restorations with predictable morphology (14, 15). Several matrix-based approaches have been proposed to standardize palatal morphology, yet each carries limitations that compromise its use in multiple-diastema cases. Silicone indices, while accurate in reproducing palatal anatomy from a diagnostic wax-up, are opaque; this prevents visual monitoring of composite adaptation, air-void formation, and shade layering during placement, so errors are only detected after removal (16). 3D-printed matrices improve the reproducibility of the palatal shell but require digital planning, printing time, and additional cost, making them less accessible for single-visit or resource-limited settings, and they remain rigid, offering no interproximal adaptability once the diastema is closed (12, 13, 16). Conventional celluloid strips are transparent and low-cost, but lack rigidity and dimensional stability, allowing distortion during freehand palatal sculpting; this makes final contour highly operator-dependent, with a greater risk of over- or under-contouring, flat or bulky emergence profiles, and inconsistent interproximal embrasure problems that are magnified when several adjacent diastemas require simultaneous, symmetric closure. None of these techniques alone, therefore, combines transparency for real-time visual control, rigidity for accurate palatal contour, and adaptability for interproximal/emergence profile management (16).

This technique addresses the lack of a single matrix system that allows direct visualization, dimensional stability, and controlled pre-curved interproximal contour simultaneously, particularly at diastema-adjacent sites, where emergence-profile errors are most visually and functionally consequential. This combination can be unique for the closure of anterior diastemas, where morphological control is most crucial. This procedure is not limited to diastema cases; it can be used for composite veneer rehabilitation of any accessible tooth, particularly in the esthetic zone, though sites adjacent to a diastema benefit most. The clinical efficacy of this treatment was assessed after 1 week and after 12 months in 20 proximal sites (10 restorations), using conventional modified United States Public Health Service (USPHS) and Fédération Dentaire Internationale (FDI) Clinical Criteria to enable a comprehensive outcome assessment in accordance with existing research and case reports (17, 18). It is hypothesized that combining a liquid dam-stabilized transparent palatal matrix with a pre-curved, pull-through interproximal celluloid matrix would allow more predictable control of palatal contour and interproximal emergence profile than conventional single-matrix techniques, and this case report describes the technique and reports its 1-week and 12-month clinical outcomes.

## 2 Case description

### 2.1 Clinical technique overview: the dual-matrix approach

**Figure 1:**
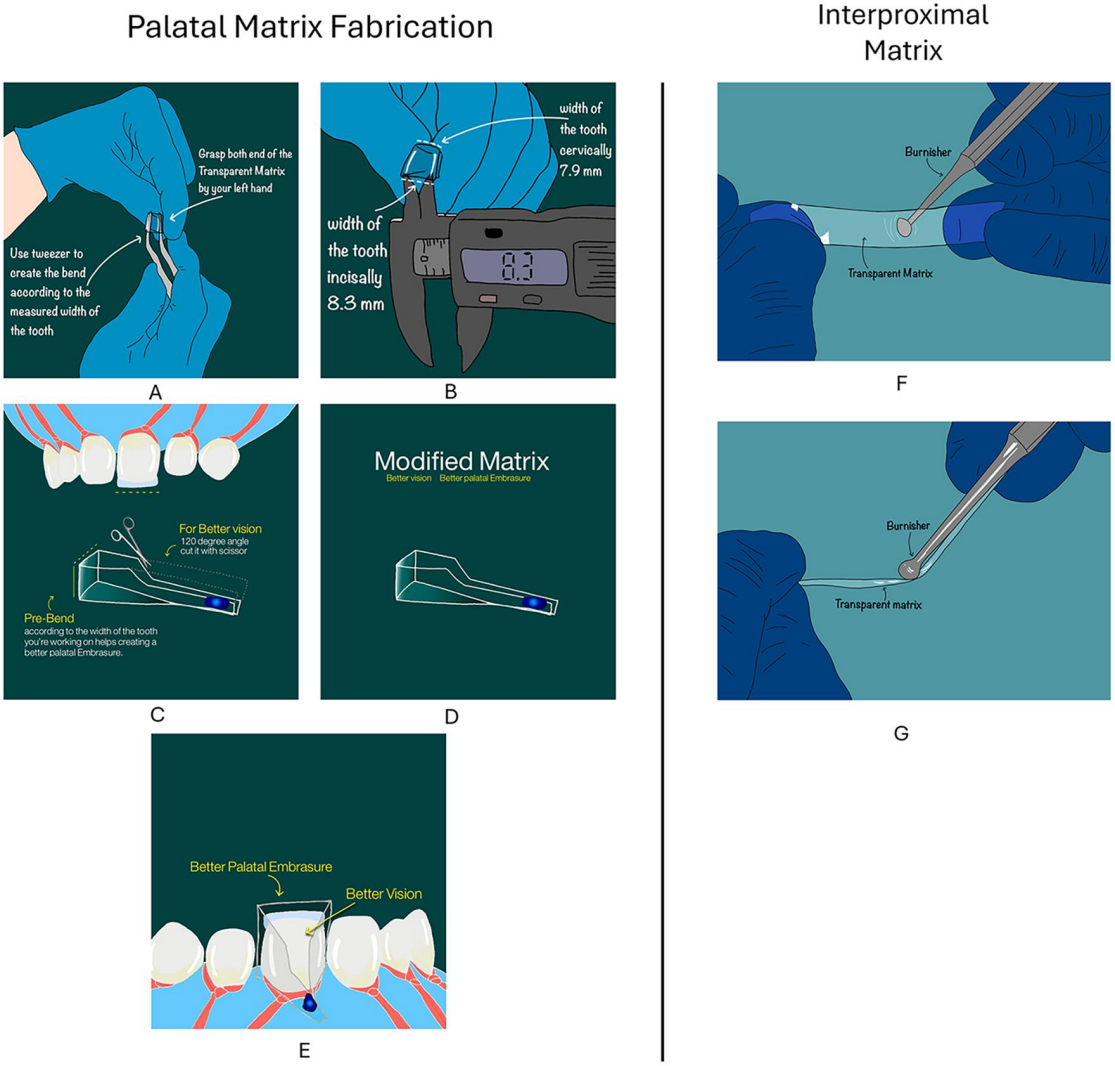
Matrix fabrication. **(A–E)** Palatal matrix: **(A)** Transparent strip sized to mesiodistal tooth width. **(B)** Incisal and cervical widths measured with digital calipers. **(C,D)** A ∼120° angular cut improves visibility and creates the palatal embrasure area. **(E)** Final customized matrix ready for composite application. **(F,G)** Interproximal matrix: **(F)** Occlusal and **(G)** lateral views of the pre-curved “C” configuration that guides composite for controlled mesiodistal thickness and emergence profile.

The dual-matrix approach (Figures 1A–G) requires the use of two matrices. The first matrix is designed for making the palatal shell. The second matrix contours the interproximal walls and defines the emergence profile between adjacent teeth. Supplementary Table S1 summarizes the minimum instruments and materials.

#### 2.1.1 Palatal matrix: palatal shell creation

A transparent film (PD, Vevey, Switzerland), 8 mm or wider depending on tooth size, allowed continuous visualization of contour, emergence profile, and incisal edge position during sculpting; the 8 mm width fully covers the palatal surface of an average incisor in one sheet, avoiding repositioning, with wider strips used for larger teeth. Unlike celluloid strips, which are similarly transparent but lack rigidity for freehand sculpting, this matrix, once stabilized retains a fixed shape that transfers directly onto the palatal surface, reducing operator-dependent variability.

The matrix was stabilized extraorally by applying a liquid dam (Cerkamed, Stalowa Wola, Poland) to both ends at 1.5–2 mm thickness and light-curing for 20 s per manufacturer specification; once cured, the liquid dam rigidly held the celluloid band in place as a single unit. The stabilized matrix was then trimmed extraorally at an angle before being transferred intraorally onto the palatal surface (Figures 1A–E). The liquid dam serves two roles: it rigidifies the matrix after light-curing, enabling direct transfer to the palatal surface, and it acts as a barrier confining the bonding agent to the operative field, limiting adhesive spread onto adjacent unprepared tooth structure. Rubber dam isolation (Section 2.2.2) was used throughout to maintain a dry, contamination-free field essential for optimal adhesive bond strength and composite physical properties, and by retracting gingival tissue, provided clear visualization of the cervical margin, reducing the risk of subgingival composite overhang.

A high-translucency enamel composite (Incisal HVT, Shofu, Kyoto, Japan) was applied to the matrix and sculpted to form the palatal shell, defining the first palatal wall and scaffolding the subsequent dentin and enamel layers***,*** The shell surface against the matrix cured fully polymerized, while the air-exposed surface retained a thin oxygen-inhibited layer that promoted chemical bonding of the next composite increment, requiring no additional surface treatment. Shofu composites were selected mainly for their Giomer Surface pre-reacted glass ionomers (S-PRG) filler technology, which provides sustained fluoride/ion release and acid-neutralizing capacity, offering added protection at interproximal margins where hygiene access is limited; the composite's translucency, though also achievable with other modern composites, further supported anterior shade-matching. Following light-curing (Elipar DeepCure-S, 3M ESPE, St. Paul, MN, USA; 1,470 mW/cm^2^), excess composite was removed using gentle back-and-forth motions of a metal abrasive strip.

#### 2.1.2 Interproximal matrix: pull-through contouring and proximal adaptation

To solve the interproximal profile problem, a second transparent matrix (PD, Vevey, Switzerland) was pre-curved extra-orally into a C-shape using a burnisher (Aesculap, Tuttlingen, Germany) or the flat ends of a tweezer handle, adding curvature mesiodistally to match the interproximal site (Figures 1F,G). Pre-curving allows the matrix to passively adapt to the proximal embrasure rather than relying on freehand intra-oral shaping, which is less reproducible across multiple adjacent sites. Once shaped, the matrix was inserted slightly subgingivally around the tooth and pulled gently from the facial to the palatal aspect (pull-through technique) (19), guiding composite along the proximal wall to establish the emergence profile and proximal contact (Supplementary Video 1). The proximal area was refined relative to the adjacent tooth using a thin modelling instrument (Modella, LM Dental, Parainen, Finland).

### 2.2 Clinical case presentation

#### 2.2.1 Patient information and treatment planning

The case report was written in accordance with the CARE guidelines. The patient signed the bilingual institutional consent form and provided written informed consent for all clinical procedures, including photography and dissemination.

**Figure 2:**
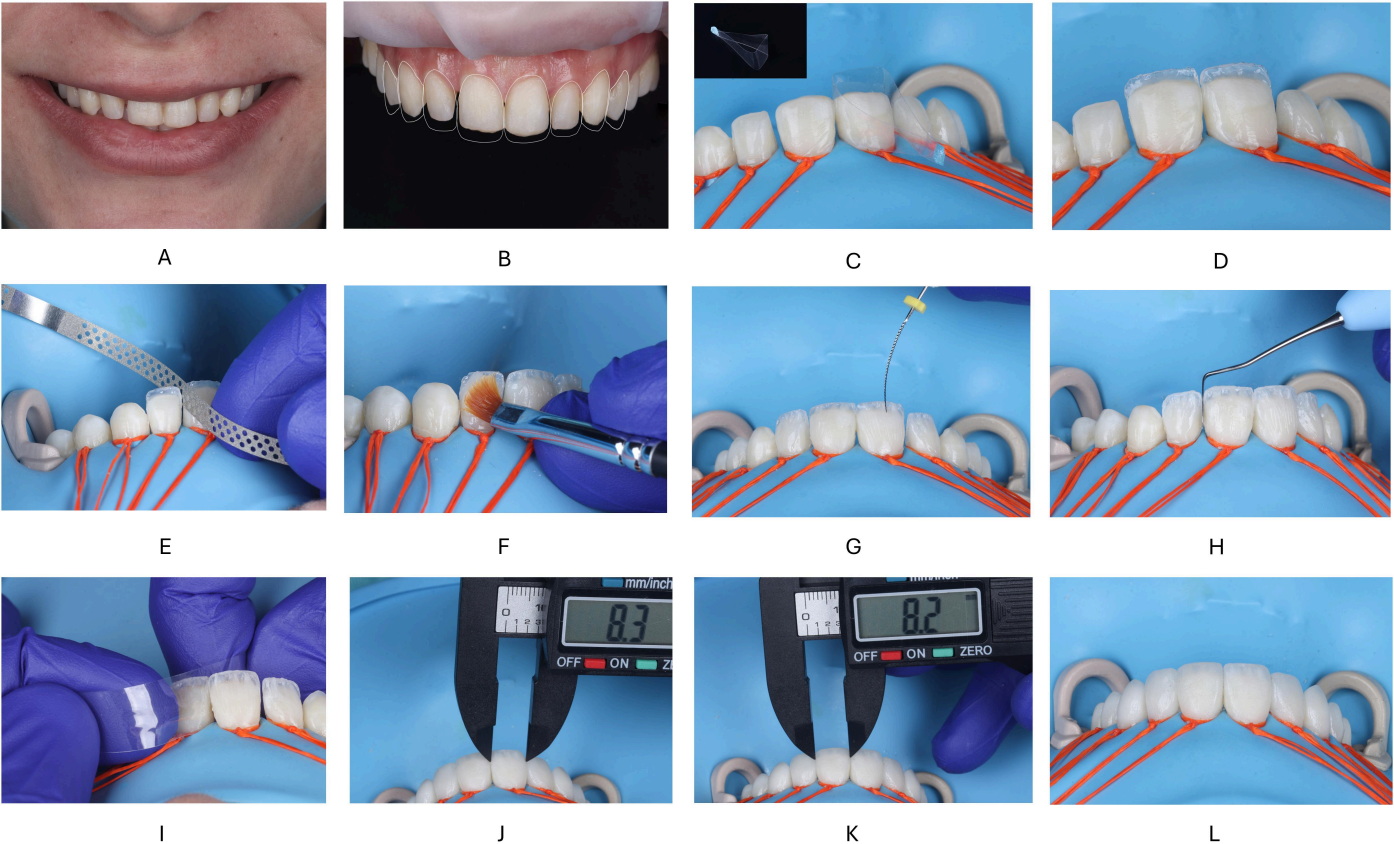
Step-by-step direct composite rehabilitation of maxillary anterior diastemas. **(A)** Initial smile. **(B)** Digital Smile Design (DSD). **(C,D)** Palatal shell with a clear palatal matrix. **(E)** Interproximal finishing. **(F)** The dentine composite stacking stage. **(G)** The Mamelon creation stage. **(H)** Proximal contouring step. **(I)** The use of the “pull-through palatal matrix step” allows the creation of contact areas and the correct emergence profile. **(J,K)** Checking tooth proportions, particularly height and mesiodistal width ratios. **(L)** The end of the restoration phase.

A 22-year-old female patient presented for esthetic treatment of bilateral maxillary anterior peri-central diastemas. The medical, periodontal, and oral hygiene examinations were within normal limits. No relevant family, genetic, or psychosocial history was reported, and no prior restorative or orthodontic treatment had been performed on the affected teeth. On clinical examination with the FDI two-digit notation method, a diastema was seen between the teeth 12 and 11 (right lateral and central incisors) and 21 and 22 (left central and lateral). The Patient had no history of smoking or parafunctional habits. Preoperative smile design was based on assessment of tooth form, alignment, proportions, incisal edge location, and sizes (Figure 2A). The patient chose direct Composite Veneer bonding instead of Ceramic Laminate veneer placement and orthodontic therapy with space closure. The selection was made based on the age of the patient, the need to preserve the enamel substrate, and the possibility of a reversible single-visit solution (20, 21). The intervention was a single-visit direct composite veneer restoration (teeth 15–25) under rubber dam isolation; no intraoperative plan changes occurred, and the only post-treatment deviation was the unplanned chairside repair at 12 months (Section 3.3).

#### 2.2.2 Digital planning and operative field isolation

A two-dimensional Digital Smile Design (DSD)-inspired template was created using presentation software (PowerPoint 2021, Microsoft, Redmond, WA, USA) for preliminary visualization of the proposed restorative outcome (Figure 2B). Rubber dam isolation (Cranberry, Pinole, CA, USA) was accomplished by using dental floss (TePe, Malmo, Sweden) for interdental adaptation and KSK clamps (KSK, Tokyo, Japan) on the maxillary first molars for anchoring.

#### 2.2.3 Restorative procedure: stratified layering and contouring

The dual-matrix technique outlined in Section 2.1 was employed to position composite veneers with palatal shells and pull-through interproximal contouring on all ten maxillary teeth from tooth 15 to tooth 25. The technique was especially valuable at the diastema-adjacent sites (teeth 11, 12, 21, 22), where emergence-profile control and palatal shell stability were most critical. Palatal shells were built for each central incisor following the technique described in Section 2.1.1 (Figures 2C,D). After polymerization, excess composite was removed interproximally using a diamond stainless-steel strip (Maxill, Canada) (Figure 2E).

A dentin-shade composite (Beautifil II LS A1, Shofu, Kyoto, Japan) was applied from the mid-cervical third to the incisal edge, with mamelon morphology incorporated within this layer, sculpted using a composite brush (Compo Brush, Smile Line, St. Imier, Switzerland) (Figure 2F). Prior to polymerization, mamelon anatomy was delineated using a size #15 endodontic file (Dentsply Sirona, Charlotte, NC, USA) and a fine fissure instrument (Figure 2G). A thin modeling instrument (Modella, LM Dental, Finland) adapted the composite along proximal surfaces (Figure 2H), followed by the pull-through technique of the modified interproximal matrix (Figure 2I). Digital caliper measurements (Mitutoyo, Kawasaki, Japan) confirmed balanced tooth dimensions: tooth 11: 8.3 mm; tooth 21: 8.2 mm (Figures 2J,K). A dentin-shade composite (Beautifil II LS B1, Shofu, Kyoto, Japan) was used to create the incisal halo effect (Figure 2L). The complete stratification sequence is illustrated schematically (Supplementary Figure 1).

#### 2.2.4 Finishing and polishing protocol

**Figure 3:**
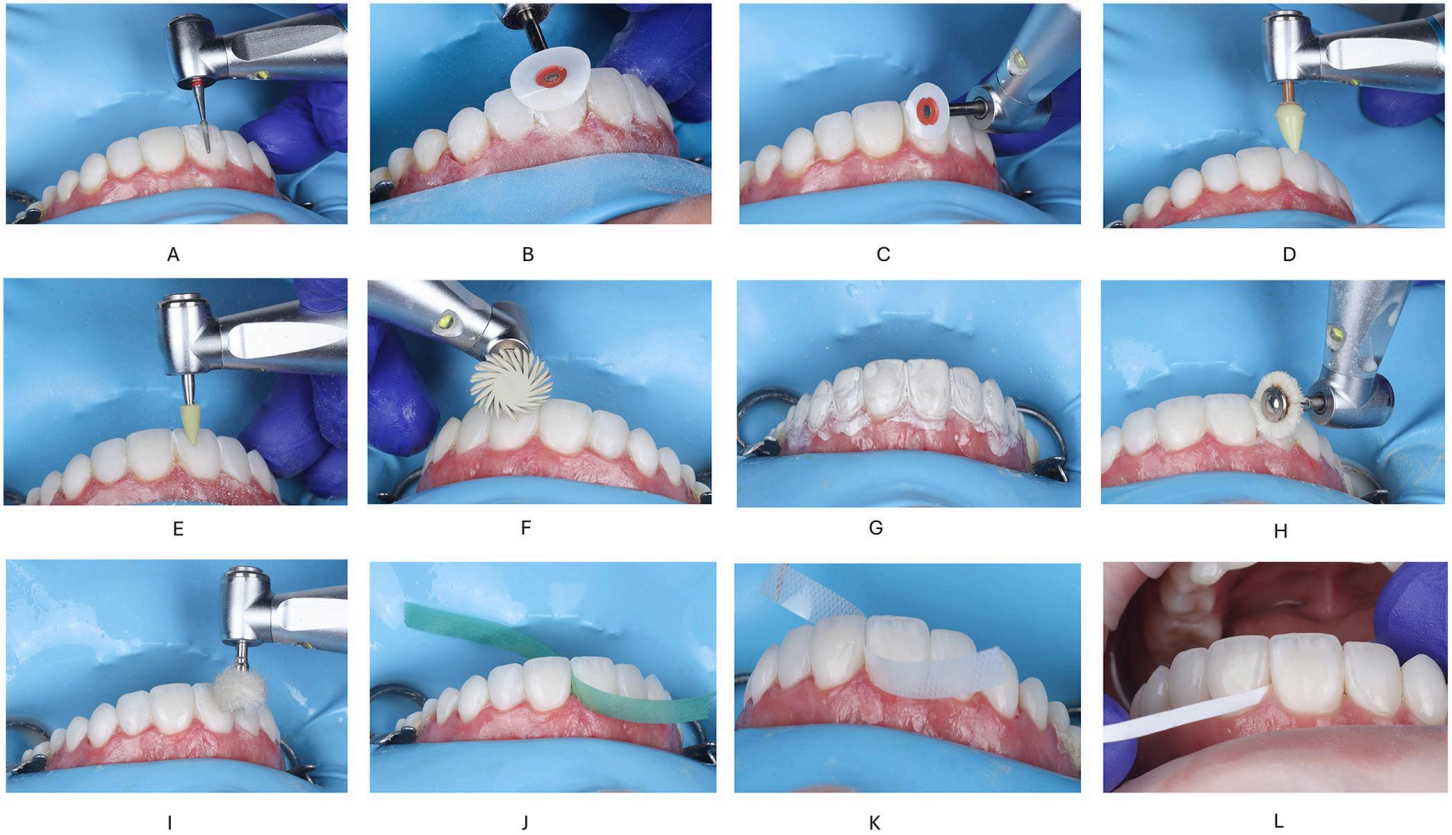
Finishing and polishing sequence for diastema closure (when composite is applied directly). **(A–E)** Contouring, anatomical characteristics, and surface texture using finishing burs and abrasive discs. **(F- I)** Final polishing was done in sequence using spiral wheels, brushes, and buffing instruments. It gave the composite an enamel-like shine. **(J- K)** The interproximal area was refined using polishing strips. **(L)** Floss confirms proximal contact and the flush appearance of the edge.

A systematic five-stage finishing and polishing protocol was followed (Figures 3A–L): basic morphology with a red-band perio bur (Intensive, Switzerland) and medium-grit disc (OptiDisc 4181, Kerr, USA) (Figures 3A,B); primary morphology with a soft finishing disc (OptiDisc 4184, Kerr, USA) (Figure 3C); secondary anatomy mapping with a cone-shaped Enhance bur (Dentsply Sirona, USA) (Figure 3D); surface texturing with a polishing bur (Jota, Switzerland) (Figure 3E); and high-gloss polishing with a spiral wheel (Jota) (Figure 3F), Prisma Gloss paste (Dentsply Sirona) (Figure 3G) with goat hair brushes (Ultradent, USA) (Figure 3H), and a fine-shine buff wheel (Henry Schein, USA) (Figure 3I). Proximal surfaces were finished and polished with medium and fine Epitex strips (GC, Japan) (Figures 3J,K), and waxed floss (TePe, Sweden) was used to verify the absence of overhangs (Figure 3L).

### 2.3 Evaluation protocol

Restorations were assessed at baseline (1 week after polymerization and adjustment) and 12-month recall. Multiple evaluation systems were used: modified USPHS (17) and FDI clinical criteria (18).

USPHS rates restorations Alpha (excellent), Bravo (acceptable), or Charlie (poor). One is clinically excellent, two is good, three is sufficient, four is unsatisfactory, and five is poor in the FDI system. Clinically acceptable scores were 1–3, unacceptable scores were 4–5. Both systems were used for complementary resolution: USPHS's simpler three-grade scale allows comparison with earlier composite veneer literature, while FDI's five-grade scale is more discriminative within the “acceptable” range. This proved clinically useful — in the tooth 12 chip event (Section 3.3), USPHS graded the outcome Bravo, but the FDI score of 3 specifically flagged sufficient severity to justify targeted repair rather than monitoring.

Functional, biological, and esthetic properties were considered: fracture and retention, marginal adaptation, wear, proximal anatomical shape, and postoperative sensitivity, secondary caries and erosion, tooth integrity, and surrounding mucosa.

M.H. and R.J., two calibrated examiners, independently performed all assessments after seeing reference pictures and clinical cases. Cohen's Kappa (*κ*) was used to calculate inter-rater agreement. Kappa was appropriate given the categorical/ordinal scoring and two independent examiners. Visual inspection and gentle tactile exploration with a dental operating microscope with integrated digital imaging (620 Pro, Mediwork, Shanghai, China) assessed marginal integrity and staining. A light explorer gently assessed surface texture. Visual inspection under dry conditions assessed color match and anatomical form. Ten maxillary teeth (15–25) and twenty proximal restoration sites were examined. Table 1 shows all clinical and treatment visits. Findings are reported relative to proximal sites (*n* = 20) where site-level tracking applies (e.g., interproximal staining), and relative to restorations (*n* = 10) for restoration-level findings (e.g., discoloration, surface luster, gingival response, chipping).

**Table 1:** Episode of care timeline. *κ* = Cohen's kappa inter-rater agreement between two independent examiners.

Time point	Visit/event	Clinical findings/interventions	Outcome/score (*κ*)
Pre-treatment	Consultation & planning	Bilateral peri-central diastemas (12–11, 21–22). Composite veneer rehab (teeth 15–25) using DSD template planning. Periodontal/medical screening and informed consent obtained. Direct composite veneers were chosen.	Treatment plan approved; documentation complete
Day 0	Single-visit veneer placement	Rubber dam. Dual matrix approach. Palatal shells + interproximal contouring by pull-through 15–25. Layered dentin/enamel (stratified) Finishing/polishing: five-step	Restorations placed; immediate esthetic satisfaction
1 week (Baseline)	Baseline evaluation (2 examiners)	Assessment by USPHS/FDI of 10 restorations (teeth 15–25) and 20 interproximal sites; two calibrated examiners; standardization session; microscopic examination.	All 10/10 sites: Alpha/FDI Score 1 (all parameters); κ = 1.00
12 months	Follow-up, evaluation & chairside repair (2 examiners)	USPHS/FDI re-examination. Bravo/FDI Interproximal Staining 2–3: 3 places (15%). Bravo/FDI 2–3 marginal discoloration: 1 location (10%). Surface gloss Bravo/FDI 2: 2 restorations (20%). Adjacent mucosa Bravo/FDI 2 1 location (10%). Incisal tooth chip 12: Bravo/FDI Score 3. Chairside repair: air abrasion (50 µm Al2O3), 37% H3PO4 etchant, silane, universal bonding, composite stratification.	10/10 clinically acceptable (FDI 1–3); minor staining/gloss loss in <30% sites; 1 incisal chip (FDI 3) – repairable; κ = 0.94

## 3 Diagnostic assessment, therapeutic intervention, follow-up, and outcomes

### 3.1 Immediate post-operative outcome

**Figure 4:**
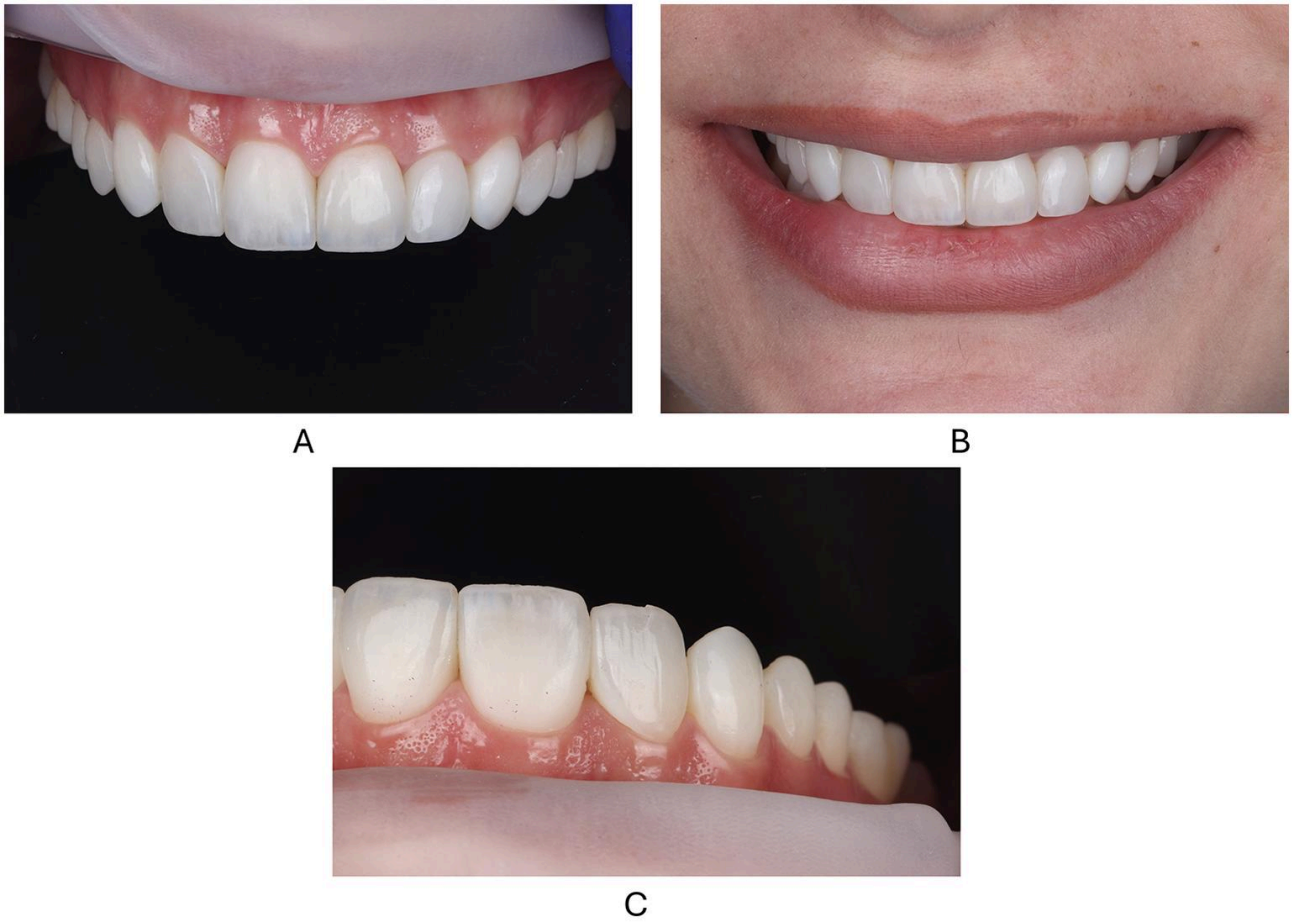
Final outcome and 12-month clinical assessment. **(A)** High-contrast (black-background) intraoral view highlighting surface texture, anatomical detail, and seamless integration with adjacent natural dentition. **(B)** Extraoral smile view showing improved tooth proportions, symmetry, and overall smile harmony. **(C)** Twelve-month follow-up oblique lateral view of right maxillary anterior veneers; the localized incisal chip on tooth 12 (white arrow) required and received chairside repair at recall.

The final smile demonstrated improved tooth proportions, symmetry, and smile harmony. Black-background contrast photography demonstrated surface texture, anatomical detail, and seamless optical and mechanical integration with adjacent natural teeth, respectively (Figure 4A). The patient was satisfied with the immediate esthetic result (Figure 4B).

### 3.2 Baseline and 12-month clinical evaluation

All 10 restorations, including the 20 interproximal sites at each time point, were rated individually by both examiners, with perfect agreement at baseline (*κ* = 1.00) and near-perfect at 12 months (*κ* = 0.94); scores were determined by consensus. At baseline, all 10 restorations received Alpha (USPHS)/Score 1 (FDI) for all metrics, establishing an appropriate baseline for later comparison. Patient-reported satisfaction was assessed verbally at each recall; the patient reported satisfaction and no discomfort or sensitivity at both visits, with the incisal chip (Section 3.3) as the only adverse event.

At baseline, all twenty proximal sites had Alpha staining. At 12 months, three interproximal sites (15%) and one restoration (10%) showed Bravo (USPHS)/FDI Score 2–3, indicating minor discoloration with progression. This is the most common early result in composite veneers (5, 6). Two restorations (20%) showed light surface dullness at interproximal margins (Bravo/FDI Score 2), and one restoration (10%) showed mild transitory gingival marginal redness (Bravo/FDI Score 2) associated with minimal plaque deposition; no treatment was necessary for either observation. Color match, postoperative sensitivity, secondary caries, and tooth integrity remained Score 1 at all sites. A localized incisal chip (Figure 4C) on tooth 12 (10%) resulted in a drop to Bravo (USPHS)/FDI Score 3 (clinically sufficient/acceptable) at 12 months. Although both systems deemed the chip acceptable, the FDI Score 3 better captured its severity, enabling a confident in-office repair rather than full replacement. 1 restoration (10%) was Bravo/FDI Score 2 for surface texture due to the localized roughness caused by the chipping. The results are all shown in Table 2.

**Table 2:** Clinical examination of restorations at baseline (1 week post-operative) and 12-month follow-up using modified USPHS and FDI clinical criteria.

Evaluation parameter	USPHS baseline (1 wk)	USPHS 12 months	FDI baseline (1 wk)	FDI 12 months
Esthetic properties				
Surface luster(a)^b^	Alpha (10/10)	Alpha (8/10) Bravo^b^ (2/10) [20%]	Score 1 (10/10)	Score 1 (8/10) Score 2^b^ (2/10) [20%]
Interproximal staining(b)	Alpha (20/20)	Alpha (17/20) Bravo (3/20) [15%]	Score 1 (20/20)	Score 1 (17/20) Score 2–3 (3/20) [15%]
Marginal discoloration(a)^c^	Alpha (10/10)	Alpha (9/10) Bravo (1/10) [10%]	Score 1 (10/10)	Score 1 (9/10) Score 2–3 (1/10) [10%]
Color match & translucency(a)	Alpha (10/10)	Alpha (10/10)	Score 1 (10/10)	Score 1 (10/10)
Esthetic anatomical form(a)	Alpha (10/10)	Alpha (9/10) Bravo^a^ (1/10) [10%]	Score 1 (10/10)	Score 1 (9/10) Score 3^a^ (1/10) [10%]
Functional properties				
Fracture & retention(a)	Alpha (10/10)	Alpha (10/10)	Score 1 (10/10)	Score 1 (10/10)
Marginal adaptation(a)^c^	Alpha (10/10)	Alpha (10/10)	Score 1 (10/10)	Score 1 (10/10)
Wear(a)	Alpha (10/10)	Alpha (10/10)	Score 1 (10/10)	Score 1 (10/10)
Biological properties				
Postoperative sensitivity(a)	Alpha (10/10)	Alpha (10/10)	Score 1 (10/10)	Score 1 (10/10)
Secondary caries/erosion(a)	Alpha (10/10)	Alpha (10/10)	Score 1 (10/10)	Score 1 (10/10)
Adjacent mucosa(a)^e^	Alpha (10/10)	Alpha (9/10) Bravo^e^ (1/10) [10%]	Score 1 (10/10)	Score 1 (9/10) Score 2^e^ (1/10) [10%]
Surface texture(a)^d^	Alpha (10/10)	Alpha (9/10) Bravo (1/10)	Score 1 (10/10)	Score 1 (9/10) Score 2–3 (1/10)

**USPHS criteria:  **Alpha, clinically acceptable; Bravo, clinically acceptable; Charlie, clinically unacceptable.  **FDI criteria:  **Score 1, clinically outstanding; Score 2, clinically good; Score 3, clinically sufficient/acceptable; Score 4, clinically unsatisfactory; Score 5, clinically poor/unacceptable. All statistics are reported as the number of restorations or sites/number of restorations or sites investigated; percentages refer to non-ideal scores. **(a) **Per restoration (*n* = 10). **(b) **Evaluation of each interproximal surface – mesial and distal per restoration (*n* = 20).

a Esthetic anatomical form: one restoration (tooth 12) scored Bravo/FDI Score 3 due to an isolated repairable incisal chip. Reviewed at 12 months. Corrected.

b Surface luster: two restorations (20%) showed mild dullness at interproximal borders (Bravo/FDI Score 2) due to limited access for polishing.

c Marginal discoloration and marginal adaptation were evaluated in all 10 restorations.

d Surface texture: tooth 12 showed loss of incisal edge at 12-month follow-up, which caused localized roughness; The score is based on the pre-repair condition.

e Adjacent mucosa: transient, slight marginal erythema (Bravo/FDI Score 2) due to slight plaque deposition was observed adjacent to one restoration (10%). No treatment required.

### 3.3 Chipping event and chairside repair

Patient reported accidental biting on a hard item between appointments, causing the localized incisal chip on tooth 12 (Figure 4C). Neither discomfort nor sensitivity occurred. The recall session with rubber dam isolation solved the problem instantly, with an FDI score of 3 at the 12-month checkup. Chipped margin edges were shaped. Aluminum oxide 50-micron particles were air-abraded. A rinse and air drying followed 30 s of 37% phosphoric acid gel etching. Silane and a universal bonding agent were applied to the repair surface. A thin layer of dentin-shade composite (Beautifil II LS B1, Shofu, Japan) was used to reconstruct the incisal halo, followed by enamel shade composite to rebuild the contour. Bonding and repair were integrated into optical continuity. The repair was done without local anesthesia. The finishing and polishing sequence was changed in Section 2.2.4.

## 4 Discussion

Baseline clinical performance was recorded according to USPHS and FDI standards, along with repeated measures at 12 months, to facilitate adequate assessment of the results, which allows the direct comparison with other direct-veneer investigations. All veneers were scored Alpha or FDI Score 1 in all categories at baseline condition, which indicated maximum quality from the outset, setting a definite benchmark. Without that baseline, the restoration quality alteration assessment at 12 months would not be possible.

Several alternative techniques address these same challenges with different trade-offs (Supplementary Table S2). Silicone putty matrices are stable but limit visibility, though clear variants improve this at added cost and time (16). Celluloid strips are translucent but lack rigidity for unsupported palatal shell formation. Injectable composite techniques enable rapid, low-shrinkage placement but limit control over internal layering and mamelon detail. Prefabricated veneer systems reduce chair time but adapt poorly to irregular diastema widths (14, 15). 3D-printed matrices offer high reproducibility but require digital infrastructure and remain rigid once fabricated (12, 13). The dual-matrix technique combines visibility, stability, and interproximal adaptability not offered by any single existing method. However, none of these approaches fully eliminate technique sensitivity, and the dual-matrix technique should be considered a method that redistributes, rather than removes, procedural complexity across multiple steps. Therefore, the selection of technique should be guided not only by material properties but also by operator experience and case-specific anatomical considerations.

The minor staining, surface dullness, and gingival redness observed at 12 months (Section 3.2) are consistent with the most frequently reported early complications for composite veneers, particularly at interproximal margins where finishing access is limited (5, 6), and reinforce the importance of proximal hygiene reinforcement at recall. These findings may also reflect early marginal degradation and increased surface roughness associated with composite materials, which can promote plaque accumulation and compromise long-term esthetic stability if not properly maintained. One major advantage that composites have over ceramics is reparability, as demonstrated by the effective chairside repair performed without complete replacement of the restoration (22). However, this advantage may also mask underlying material degradation, as repeated repairs can alter surface integrity and potentially affect long-term color stability and wear resistance. The chipping event, although successfully repaired chairside, illustrates a durability trade-off inherent to composite veneers relative to ceramic: composite's lower flexural strength and fracture toughness make it more susceptible to mechanical damage from parafunctional or accidental loading, even when bonding and marginal adaptation remain otherwise sound (1, 6). Importantly, this event does not necessarily indicate failure of the dual-matrix technique itself, but rather highlights the intrinsic material limitations of direct composite restorations under functional stress. This reinforces that the long-term success of the dual-matrix technique, like other direct composite approaches, is multifactorial and depends not only on operative execution but also on patient-related factors, including dietary habits, oral hygiene, and parafunctional activity, which may significantly influence restoration longevity. Accordingly, appropriate case selection, patient education, and regular maintenance should be considered essential components for optimizing long-term outcomes when using this technique.

Direct composite veneers were chosen for their conservative, enamel-preserving nature appropriate to the patient’s young age; while an excellent long-term alternative to indirect restorations, they may eventually require indirect replacement later in life (20, 21).

Limitations of the study are the availability of only 12 months of follow-up, which may not sufficiently assess the long-term color stability or fracture resistance of the composite veneers. Systematic assessments of resin composite laminate veneers have reported moderate survival rates from 24 to 97 months (4). Additional follow-up is necessary, with adequate reporting using standardized criteria, so that the limitations of new techniques and material properties can be systematically analyzed. The external validity of the scoring was restricted by the fact that calibration was conducted specifically to establish inter-rater reliability between the two operators and was not benchmarked against a previously validated gold-standard examiner, despite the fact that examiners were trained using standardized reference photographs. The single case design does not permit generalization to other operators or patient groups. The technique carries a significant operator-dependent learning curve, as achieving consistent palatal shell thickness and controlled interproximal adaptation requires precise handling and sequencing. In less experienced hands, inaccuracies in matrix positioning or composite manipulation may lead to over-contouring, marginal discrepancies, or suboptimal interproximal adaptation, potentially compromising clinical outcomes. As with any single-case report, twelve-month outcomes cannot be extrapolated to predict longer-term performance, particularly wear and fracture resistance, which typically become more apparent beyond one year. These limitations may affect both the internal and external validity of the findings, and therefore the results should be interpreted with caution when extrapolating to broader clinical settings.

## 5 Conclusions

The dual-matrix technique (liquid-dam-stabilized transparent palatal matrix + pre-curved interproximal celluloid matrix for pull-through technique) provided a controlled emergence profile and predictable palatal shell construction in the current clinical scenario with the presence of multiple diastemas. The technique may provide a cost-effective, one-visit alternative to completely freehand, other unmodified matrix-led methods, or the silicone mold technique. Longer follow-up and multi-operator studies are needed.

## Patient perspective

The visible gaps between my front teeth had impacted me psychologically for a long period of time, so I avoided smiling in critical periods of my life. I was able to talk this through with the dentist, who reassured me that the gaps could be fixed in one appointment with minimal tooth alterations. I was surprised and could not believe it for a while, but I made my decision to do it. I felt comfortable and at peace during the procedure. The results were convincing straight off, with almost perfect proportions and color enhancement that I had expected. One year later, a small chip was repaired during a private visit. I am happy with the beautiful results and easy repairability of the restorations.

## Data Availability

The original contributions presented in the study are included in the article/Supplementary Material, further inquiries can be directed to the corresponding authors.

## Supplementary material

Supplementary Video S1 Clinical demonstration of matrix pre-curving and the pull-through interproximal contouring maneuver.

Supplementary Table S1 Complete materials and instruments used in this case are listed for reproducibility.

Supplementary Table S2 Comparison of available matrix and non-matrix approaches for direct composite veneer placement in anterior diastema cases.
