# Supplementary material for: A dual-matrix technique for direct composite veneers using liquid dam and pull-through contouring: a case report
Source: Front Dent Med. 2026 Jul 30;7:1915125. doi: 10.3389/fdmed.2026.1915125 (PMC13467749; doi:10.3389/fdmed.2026.1915125)
Supplement: Supplementary file 1 [file Table1.docx]

Supplementary Material

## 1.1 Supplementary Tables

**Supplementary Table S1.** Essential materials and instruments used in this case are listed for reproducibility.

| **Material / Instrument** | **Brand / Manufacturer** | **Intended Use** |
| --- | --- | --- |
| Transparent matrix strip | PD, Vevey, Switzerland | Palatal shell & interproximal matrix |
| Liquid dam | Cerkamed, Stalowa Wola, Poland | Matrix stabilization |
| Enamel composite (palatal shell) | Incisal HVT, Shofu, Kyoto, Japan | Palatal wall formation |
| Dentin composite | Beautifil LS A1, Shofu, Kyoto, Japan | Stratification |
| Dentin composite | Beautifil LS B1, Shofu, Kyoto, Japan | Incisal halo effect |
| Light-curing unit | Elipar DeepCure-S, 3M ESPE, St. Paul, MN, USA (1470 mW/cm²) | Polymerization |
| Burnisher | Aesculap, Tuttlingen, Germany | Matrix pre-curving |
| Thin modeling instrument | Modella, LM Dental, Parainen, Finland | Proximal contouring |
| Composite brush | Compo Brush, Smile Line, St. Imier, Switzerland | Composite adaptation |
| Diamond stainless-steel strip | Maxill, Ontario, Canada | Interproximal excess removal |
| Rubber dam | Cranberry, Pinole, CA, USA | Operative field isolation |
| Digital calipers | Mitutoyo, Kawasaki, Japan | Proportional measurement |
| Microscope/imaging system | 620 Pro, Mediwork, Shanghai, China | Evaluation under magnification |

**Supplementary Table S2.** Comparison of available matrix and non-matrix approaches for direct composite veneer placement in anterior diastema cases.

| **Criterion** | **Freehand** | **Conv. celluloid** | **Silicone index** | **3D-printed index** | **Dual-matrix (present study)** |
| --- | --- | --- | --- | --- | --- |
| Palatal visualization | Limited | Partial | None — opaque | Good — translucent | Excellent — clear |
| Matrix stability | None | Low | High | High | High (liquid dam) |
| Emergence profile control | Operator-dependent | Limited | Limited | Good | Excellent (pull-through) |
| Multiple diastemas | Challenging | Challenging | Moderate | Good | Good |
| Material cost | Low | Low | Moderate | High | Low |
| Learning curve | High | Moderate | Moderate | Moderate | Moderate |
| Single visit | Yes | Yes | Yes | No — lab step required | Yes |

## 1.2 Supplementary Figures

**Supplementary Figure 1.** Schematic illustration of the complete stratified layering sequence for the Dual-Matrix direct composite veneer technique.

## 1.3 Supplementary Video

**Supplementary Video S1:** Clinical demonstration of matrix pre-curving and the pull-through interproximal contouring maneuver
